# Establishment of a risk model correlated with metabolism based on RNA-binding proteins associated with cell pyroptosis in acute myeloid leukemia

**DOI:** 10.3389/fonc.2022.1059978

**Published:** 2022-11-17

**Authors:** Ting Bin, Chao Lin, Fang-Jie Liu, Ying Wang, Xiao-Jun Xu, Dong-Jun Lin, Jing Tang, Bo Lu

**Affiliations:** ^1^ Department of Haematology, The Seventh Affiliated Hospital, Sun Yat-Sen University, Shenzhen, China; ^2^ Department of Pediatrics, The Seventh Affiliated Hospital, Sun Yat-Sen University, Shenzhen, China

**Keywords:** acute myeloid leukemia, pyroptosis, prognostic model, RNA-binding protein, metabolism

## Abstract

**Background:**

RNA-binding protein (RBP) regulates acute myeloid leukemia (AML) by participating in mRNA editing and modification. Pyroptosis also plays an immunomodulatory function in AML. Therefore, this study aimed to identify pyroptosis-related RBP genes that could predict the prognosis of AML patients.

**Methods:**

AML related expression data were downloaded from the UCSC website and Gene Expression Omnibus (GEO) database. Pyroptosis-RPB-related differentially expressed genes (PRBP-DEGs) were conducted with a protein-protein interactions (PPI) network to screen out the key PRBP-DEGs, based on which a risk model was constructed by Cox analysis, and evaluated by plotting Receiver operating characteristic (ROC) curves and survival curves. Independent prognostic analysis was performed and a nomogram was constructed. Finally, enrichment analysis was performed for high and low risk groups.

**Reuslts:**

A total of 71 PRBP-DEGs were obtained and a pyroptosis-RPB-related risk model was constructed based on IFIT5, MRPL14, MRPL21, MRPL39, MVP, and PUSL1 acquired from Cox analysis. RiskScore, age, and cytogenetics risk category were identified as independent prognostic factors, and the nomogram based on these independent prognostic factors could accurately predict 1-, 3- and 5-year survival of AML patients. Gene set enrichment analysis (GSEA) showed that the high-risk and low-risk groups were mainly enriched in metabolic- and immune-related processes and pathways.

**Conclusion:**

In this study, a risk score model correlated with metabolism based on RNA-binding proteins associated with cell pyroptosis in acute myeloid leukemia was established, which provided a theoretical basis and reference value for therapeutic studies and prognosis of AML.

## Introduction

Acute myeloid leukemia (AML) is a clonal malignant proliferative disease of myeloid primitive cells in the hematopoietic system, and it is highly heterogeneous. AML can be transformed into malignant changes of hematopoietic progenitors at different stages of normal myeloid cells (1). Currently, the treatment of AML mainly includes chemotherapy, biologics, and hematopoietic stem cell transplantation (HSCT) (2), but about 70% of patients who achieve remission will eventually relapse or evolve into refractory leukemia, leading to treatment failure and death (3). The prognosis and survival rate of AML prognosis are unsatisfactory, and it has been reported the 5-year overall survival (OS) ratein young AML patients is less than 50%, and the 2-year OS rate in older patients after diagnosis is only 20% (4).

Pyroptosis, also known as cell inflammatory necrosis, is a programmed cell death (5), It is mainly manifested as the cell membrane rupture, leading to the release of cell contents and then activation of strong inflammatory response (6). Pyroptosis plays an important role in the fight against infection, and it is widely involved in the development of infectious diseases and nervous system-related diseases (7). Moreover, Johnson et al. found that small-molecule inhibitors of the serine dipeptidases DPP8 and DPP9 (DPP8/9) induced-pyroptosis in mouse and human monocytes and macrophages for treatment of AML, it also shown that there is a strong correlation between pyroptosis and antileukemic therapy (8).

RNA-binding proteins (RBPs) are a general term for a group of proteins that perform their functions by specifically binding to RNA. To date, the human genome-wide screen has identified 1,542 RBP genes, accounting for 7.5% of all egg and white matter-encoding genes (9). RBP plays a crucial role in processes such as RNA maturation, transport, localization and translation, and it is also vital in gene expression and maintenance of genomic integrity (10, 11). Currently, Kharas et al. found that RBPs of the musashi-2 regulates normal hematopoiesis and promotes aggressive myeloid leukemia, it may be as a new prognostic marker and target for therapy in AML. However, there are still few reports on the relationship between pyroptosis and RBPs (12).

Cell pyroptosis is closely related to RBPs. Mast cells can identify the nucleic acid fragments (DNA or RNA), bacterial cell wall components (LPS), and flagella of these pathogenic microorganisms, thus stimulating immune measures, which can lead to the pathogen elimination by immune cells (13). However, there was no report on the RBPs associated with cell pyroptosis in patients with AML in public database. In this study, the RBP genes related to cell pyroptosis in AML patients were studied by bioinformatics analysis methods, and they were constructed and verified by the prognostic feature model, providing new ideas for clinical treatment.

## Materials and methods

### Data source

Gene expression data, survival information, and clinical information of AML patients were obtained from the UCSC Xena website (http://xena.ucsc.edu/), which has 173 AML samples and 70 normal samples, of which 161 AML samples have survival information and clinical information. The GSE37642 dataset was downloaded from the Gene Expression Omnibus (GEO) database (https://www.ncbi.nlm.nih.gov/), which comprises 136 AML samples with survival information. In addition, 33 pyroptosis-related genes (14) and 1542 RBP-related genes (RBPGs) (9) were available in the published literature.

### Identification and enrichment analysis of pyroptosis-RPB-related differentially expressed genes (PRBP-DEGs)

Differential analysis was performed on 173 AML samples and 70 normal samples in the UCSC Xena dataset using the “limma” package (15), and the threshold for DEGs was set at adj.*p*< 0.05 and |log_2_fold change (FC)| > 2. Then, Pearson’s correlation between pyroptosis-related genes and RBP genes was calculated. The Benjamini & Hochberg method was used for multiple test correction, and the RBP genes related to pyroptosis were screened according to |r| > 0.9 and q value< 0.05, and they were intersected with the above DEGs to obtain the PRBP DEGs related to pyroptosis. Finally, the Gene ontology (GO) system and Kyoto Encyclopedia of Genes and Genomes (KEGG) pathway enrichment analysis were performed for the PRBP DEGs using the “cluterProfiler” R package with a significance threshold of *p*< 0.05 (16).

### Construction of a protein-protein interactions (PPI) network

To investigate the interactions between PRBP DEGs, a PPI network was constructed for PRBP DEGs using the STRING website. The confidence was set to 0.4, and the protein interaction pairs were obtained by removing discrete proteins, and the protein network graph was constructed by Cytoscape software.

### Construction of the risk model

The 161 AML patients from the UCSC Xena datasetwere used as the training set, and a univariate Cox regression analysis was performed using the PRBP DEGs in the PPI network. Then the multivariate Cox regression analysis was performed based on the factors with *p*< 0.05 in the univariate Cox analysis to obtain prognostic biomarkers. Risk values were calculated for each patient using the formula: $Riskscore\sum_{i=1}^{n}coef\times id$ . Subsequently, 161 AML patients in the training set were divided into high and low risk groups based on the median risk values, and risk curves were plotted for the risk model. In addition, this study used the “pheatmap” package to plot biomarker expression heat maps in high and low risk groups. Finally, survival analysis was performed for the high- and low-risk groups, and ROC curves were plotted using the “survivalROC” package.

### Independent prognostic analysis and construction of a nomogram

To further investigate the prognosis of clinicopathological characteristics with the risk model, clinicopathological factors (RiskScore, cytogenetics risk category, age, platelet result count, gender, race, prior malignancy diagnoses, and prior treatment diagnoses) were included in univariate and multivariate Cox analyses to explore the independent prognosis of the risk model and clinicopathological factors. Then, the “RMS” R package was used to construct a nomogram for the risk model and other clinical factors at 1, 3, and 5 years (17). Finally, the validity of the nomogram was verified by plotting the calibration curve of the nomogram with the “survival” package.

### Functional enrichment analysis

The KEGG Pathway and GO biological process gene sets were used as enrichment backgrounds, and the high and low risk groups were used as phenotype files. Enrichment analysis was performed based on the different multiples of High-risk and Low-risk to obtain the up- and down-regulated pathways or functions involved in genes that differed between high- and low- risk groups, and the significant enrichment threshold was set at NOM P< 0.05. The top 10 enrichment results for each phenotype of GO biological process and KEGG pathway were selected and presented according to the ranking of NOM P values. In addition, the “limma” package was used to identify DEGs between high- and low-risk groups (15). The threshold of DEGs was set as adj. *p*< 0.05 and |log_2_fold change (FC)| > 1. Then, DAVID was used to analyze the GO and KEGG pathways in which the DEGs were involved. *p*< 0.05 and count > 2 were considered as significant enrichment results.

## Results

### 71 PRBP-DEGs were enriched to 107 GO and 8 KEGG pathway

**Figure 1:**
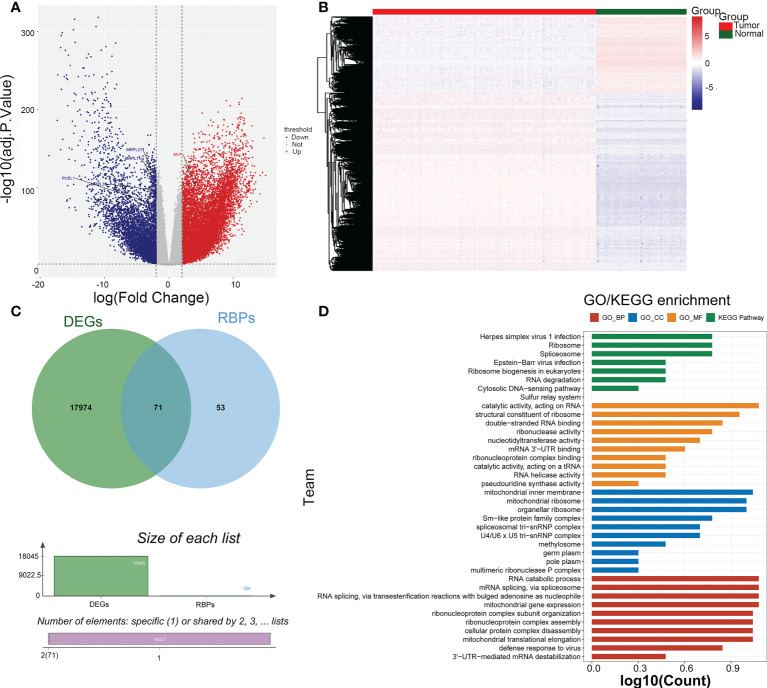
Identification of pyroptosis-RNA-binding protein (RBP)-related differentially expressed genes (PRBP-DEGs). **(A)** Volcano plot of PRBP-DEGs between AML and normal samples, the red and blue dots indicate up-regulated and down-regulated genes with adj.*p < 0.05* and |log_2_fold change (FC)| > 2 setting as criteria. **(B)** Heatmap of PRBP-DEGs in AML and normal samples. **(C)** A Venn-gram of DEGs and pyroptosis-related RPB genes. **(D)** Bar plot of Gene Ontology (GO) enrichment analysis and Genes and Genome (KEGG) pathway analysis, *p* < 0.05 were set as criteria.

There were 18045 DEGs between AML and normal samples, with 12613 up-regulated genes and 5432 down-regulated genes (**Figures 1A, B**). Pearson correlation analysis showed that 124 pyroptosis genes were associated with RBP genes. The DEGs were crossed with RBP genes associated with pyroptosis-RPB to obtain 71 PRBP-DEGs (**Figure 1C**), and they were enriched to 74 GO biological processes (GO BPs), 16 GO cell components (GO CCs), 17 GO molecular fFunctions (GO MFs), and 8 KEGG pathway, including cellular protein complex disassembly, RNA catabolic process, RNA transport, RNA destabilization, negative regulation of protein acetylation, ribonucleoprotein complex assembly, protein export from nucleus, and other protein-related pathways (**Figure 1D**; **Supplementary Tables 1**-**4**).

### Construction of a PPI network

**Figure 2:**
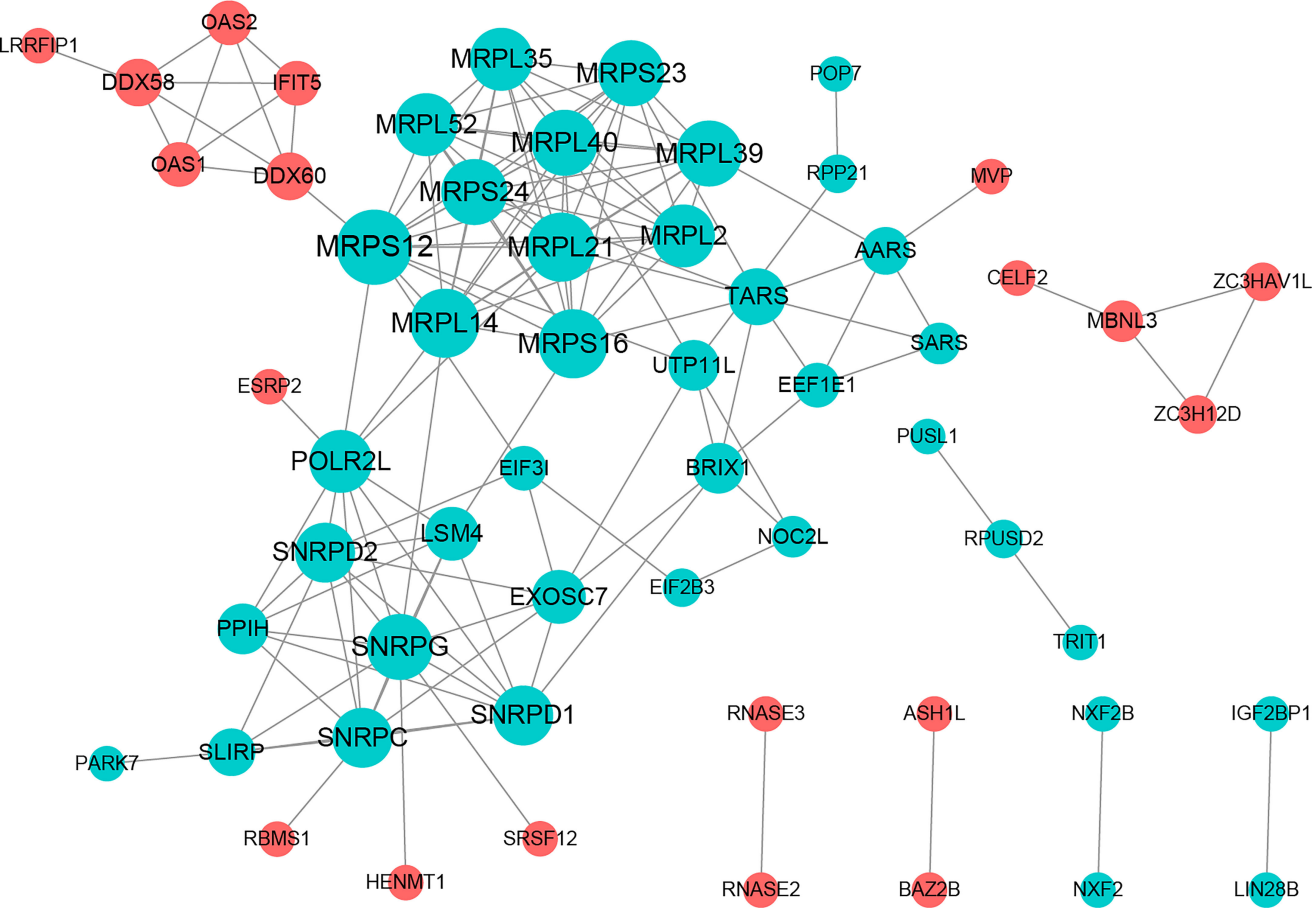
Protein-protein interactions (PPI) network constructed with PRBP-DEGs by STRING.

The PPI network included 58 nodes with 145 protein interaction pairs. The connectivity degree of MRPL40, MRPS24, MRPL21, SNRPD2, and SNRPG was high. In addition, MBNL3 was associated with ZC3H12D, ZC3HAV1L, and CELF2. MRPS12 interacts with DDX60, MRPL14, MRPL21, and MRPL39, etc. (**Figure 2**).

### Construction of the risk model

**Figure 3:**
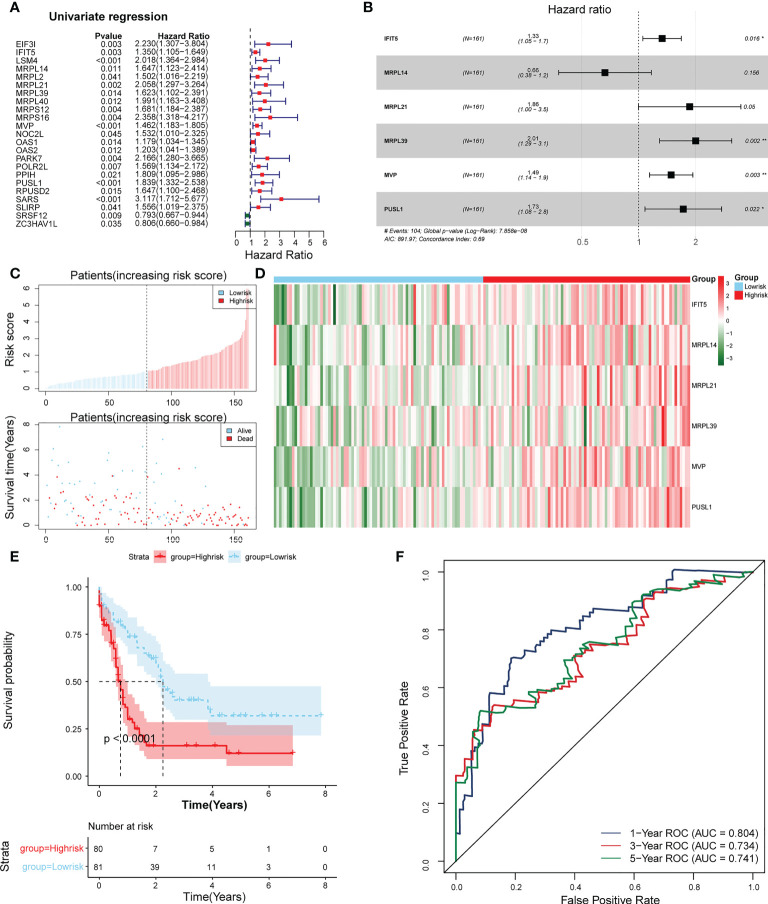
Establishment of a risk model based on 6 biomarkers. **(A)** Univariate Cox analysis of PRBP-DEGs which were selected by PPI network. **(B)** Multivariate Cox analysis to screen biomarkers. * represents p < 0.05, ** represents p < 0.01. **(C)** Distributions of risk scores and survival states between high- and low-risk groups in the training set. **(D)** Heatmap of 6 biomarkers in high- and low-risk groups. **(E)** The Kaplan-Meier survival curve for the high-and low-risk groups in the training set. **(F)** ROC curves at 1-, 3-, and 5 years in the training set.

23 PRBP-DEGs with prognostic merit were identified by univariate Cox analysis (**Table 1**; **Figure 3A**). 6 biomarkers (IFIT5, MRPL14, MRPL21, MRPL39, MVP, and PUSL1) were further detected by enrolling the 23 PRBP-DEGs in multivariate Cox analysis (**Tables 1**, **2**; **Figures 3A, B**). Among them, MRPL14 was a protective factor (HR< 1) and the rest of the genes were risk factors (HR > 1). The risk score of each sample was calculated as follows: Risk score = 0.287637545 × IFIT5 + (-0.40877001) × MRPL14 + 0.623197758 × MRPL21+ 0.697194958× MRPL39 + 0.39822836 × MVP + 0.545513896 × PUSL1, and 161 AML patients in the training set were divided into high and low risk groups by the median risk score (1.037), including 80 samples in the high-risk group and 81 samples in the low-risk group. Subsequently, we conducted principal component analysis (PCA) and t-distributed Stochastic Neighbor Embedding (tSNE) analysis on two subgroups, the results showed that the distribution of patients between high and low risk groups had clear pattern (**Supplementary Figure 1**). The risk curve was shown in **Figure 3C**. The expression of biomarkers in the high- and low-risk groups indicated that IFIT5, MRPL14, MRPL21, MRPL39, MVP, and PUSL1 were more expressed in the high-risk group (**Figure 3D**).

**Table 1:** Univariate Cox analysis to construction of the risk model.

	HR	lower.95	upper.95	p.val
SARS	3.117488934	1.712043071	5.676689692	0.00020056
PUSL1	1.838609238	1.331883653	2.538122547	0.000213762
MVP	1.461761066	1.183495432	1.80545303	0.000425672
LSM4	2.01752522	1.364048755	2.984063433	0.000440509
MRPL21	2.057589787	1.297256312	3.263561479	0.00217121
EIF3I	2.229857828	1.307191919	3.803776524	0.003249682
IFIT5	1.349731374	1.105013713	1.648644501	0.003299985
MRPS12	1.681045975	1.1839943	2.386764505	0.003680263
MRPS16	2.357846249	1.318203912	4.217434709	0.003838036
PARK7	2.166217062	1.280276367	3.665221416	0.003966997
POLR2L	1.569319071	1.134044602	2.171662686	0.006549722
SRSF12	0.793474996	0.666845724	0.944150267	0.009111989
MRPL14	1.646578327	1.123176021	2.413887171	0.010615133
MRPL40	1.990977661	1.163242997	3.407707638	0.012024053
OAS2	1.202730281	1.041115227	1.389433265	0.012168065
OAS1	1.17945007	1.034102183	1.345227282	0.013904728
MRPL39	1.623422988	1.102192997	2.391144024	0.014188686
RPUSD2	1.647408776	1.099687005	2.467934659	0.015487769
PPIH	1.808519442	1.095300701	2.98615948	0.020572574
ZC3HAV1L	0.806026539	0.65992094	0.984479719	0.034578933
SLIRP	1.555633176	1.018761732	2.375427446	0.040753344
MRPL2	1.501582438	1.016008862	2.219222591	0.041385212
NOC2L	1.532380646	1.010057548	2.324808569	0.044747457

**Table 2:** Multivariate Cox analysis to construction of the risk model.

	coef	HR	HR.95L	HR.95H	p.val
MRPL39	0.697194958	2.008111962	1.293783268	3.116838618	0.001881772
MVP	0.39822836	1.489184061	1.144212347	1.938162242	0.003056813
IFIT5	0.287637545	1.333273965	1.054794808	1.685275138	0.01611802
PUSL1	0.545513896	1.725494879	1.081634196	2.752624305	0.022063051
MRPL21	0.623197758	1.864881958	0.999043984	3.481112718	0.050352116
MRPL14	-0.40877001	0.664467036	0.377842855	1.168518698	0.155827125

**Figure 4:**
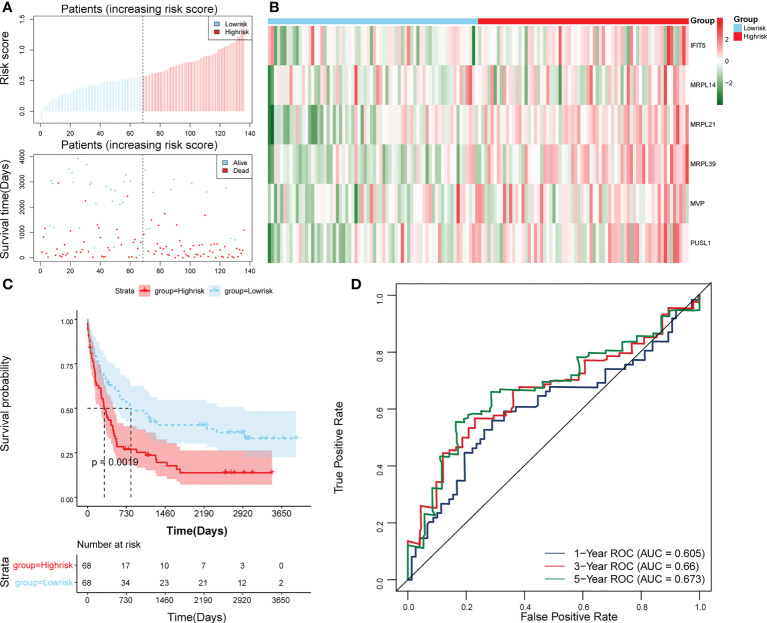
Validation of the risk model in GSE37642. **(A)** Distributions of risk scores and survival states between high- and low-risk groups in GSE37642. **(B)** Heatmap of 6 biomarkers in high- and low-risk groups in GSE37642. **(C)** The Kaplan-Meier survival curve for the high-and low-risk groups in GSE37642. **(D)** ROC curves at 1-, 3-, and 5 years in GSE37642.

The survival rate of patients in the high-risk group was lower (**Figure 3E**). The area under the curve (AUC) values for 1, 3, and 5 years were 0.804, 0.734, and 0.741 in the ROC curves, respectively, indicating that the constructed risk model could be effectively used as a prognostic model (**Figure 3F**). In addition, the GSE37642 validation set verified the applicability of the risk model. (**Figures 4A-D**)

### Independent prognostic analysis and construction of a nomogram

**Figure 5:**
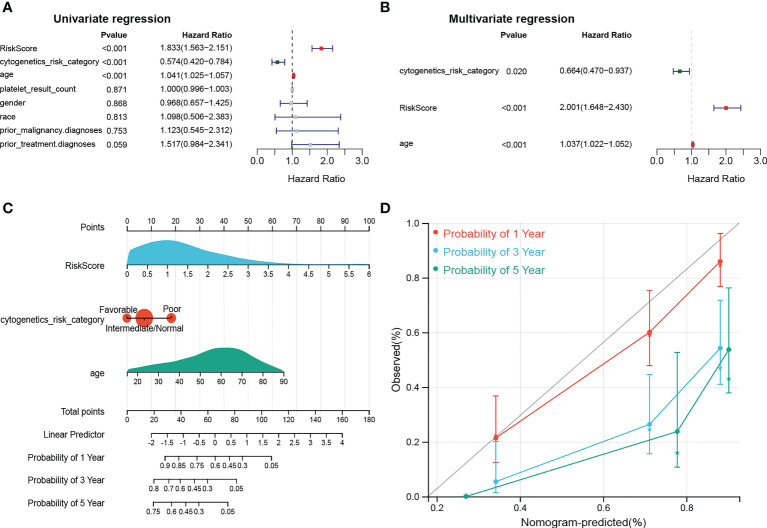
dentification of independent prognostic factors. **(A, B)** Outcomes for the univariate and multivariate Cox regression analysis of clinical parameters in AML patients (*p*< 0.05). **(C)** Nomogram of 1-, 3- and 5- year OS for AML patients. **(D)** Calibration plot of 1-, 3- and 5- year OS associated nomogram.

The results of univariate Cox analysis indicated that RiskScore, age, and cytogenetics risk category were associated with the overall survival of AML patients (*p*< 0.05), and the multivariate Cox analysis showed that RiskScore, age, and cytogenetics risk category were independent prognostic factors (**Figures 5A, B**; **Tables 3**, **4**). A nomogram using these three independent prognostic factors can predict survival at 1 year, 3 years, and 5 years (**Figure 5C**). The slope of calibration curve at 1 year was close to 1, indicating that the constructed prediction model can be used as a valid model (**Figure 5D**).

**Table 3:** Univariate Cox analysis in independent prognostic analysis.

	HR	lower.95	upper.95	p.val
RiskScore	1.833126262	1.562553294	2.150551859	0.000000000000103
cytogenetics_risk_category	0.574282951	0.420484124	0.78433617	0.000487834
age	1.041147542	1.025465494	1.05706941	0.000000191
platelet_result_count	0.999728697	0.996454031	1.003014125	0.871232716
gender	0.967616131	0.657072989	1.424926901	0.867601929
race	1.098041572	0.50600686	2.38276472	0.812957083
prior_malignancy.diagnoses	1.123048347	0.545488816	2.312123645	0.752783001
prior_treatment.diagnoses	1.517484175	0.983541135	2.34129325	0.059435784

**Table 4:** Multivariate Cox analysis in independent prognostic analysis.

	HR	lower.95	upper.95	p.val
cytogenetics_risk_category	0.663518376	0.470082914	0.936551027	0.019661517
RiskScore	2.0013394	1.648433066	2.429798016	0.00000000000239
age	1.036902937	1.021671191	1.052361769	0.00000159

### Functional enrichment analysis

**Figure 6:**
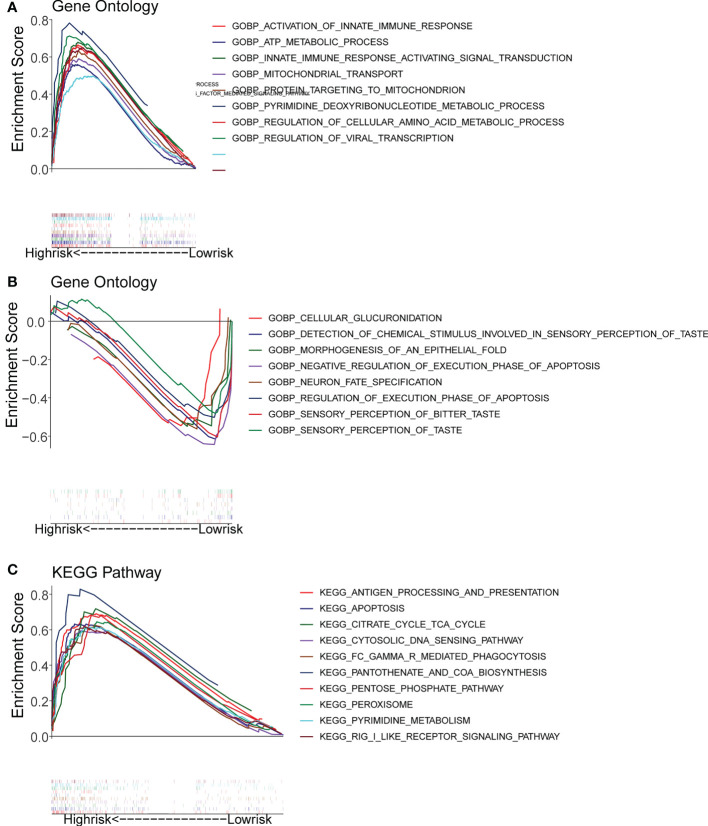
Gene Set Enrichment Analysis (GSEA) for the high- and low-risk groups. **(A, B)** GO enrichment analysis. **(C)** KEGG pathway analysis.

**Figure 7:**
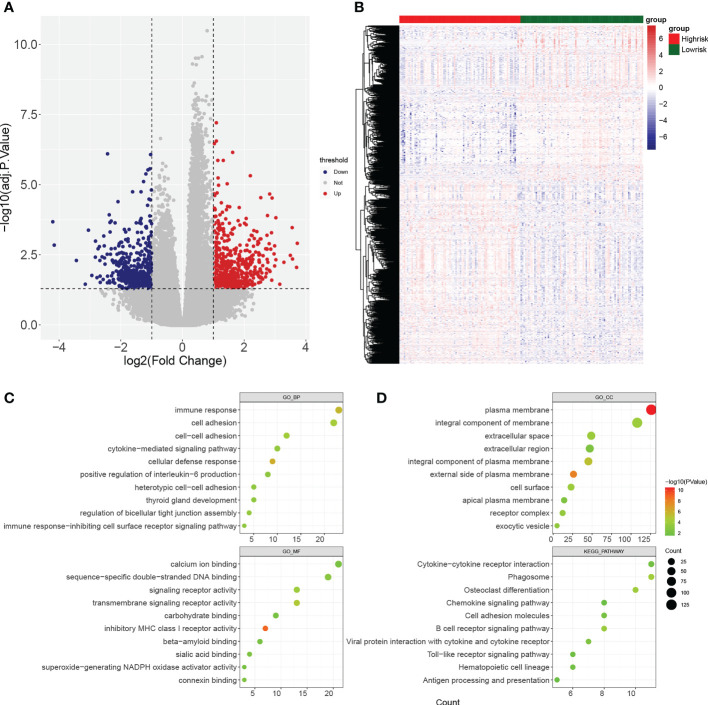
Identification of differentially expressed genes (DEGs) between high- and low-risk groups. **(A, B)** Volcano plot and heatmap of DEGs filtered with adj.p.< 0.05 and |log_2_fold change (FC)| > 1. **(C, D)** Bubble diagrams of GO enrichment analysis and KEGG pathway analysis (*p*< 0.05).

Gene set enrichment analysis (GSEA) enrichment analysis showed that the high-risk group was associated with immune functions such as activation of the innate immune response and innate immune response activating signal transduction, as well as metabolism-related processes, such as mitochondrial transport, ATP metabolic processes, regulation of cellular amino acid metabolism process, and it was also involved in antigen processing and presentation, apoptosis, and metabolism-related processes, such as citrate cycle TCA cycle and pentose phosphate pathway. The low risk group was mainly enriched in the pathways of cellular glucuronidation, negative regulation of execution phase of apoptosis, and regulation of execution phase of apoptosis (**Figures 6A-C**). Moreover, there were 997 DEGs between high and low risk groups (**Figures 7A, B**), and they were enriched to 87 GO BPs, 28 GO CCs, 23 GO MFs, and 11 KEGG pathways, mainly including immune response, immune response-inhibiting cell surface receptor signaling pathway, adaptive immune response, immune system processes, positive regulation of T cell activation and other immune-related pathways (**Figure 7C**). Thus, metabolic- and immune-related processes and pathways were closely associated with the risk model.

## Discussion

Acute myeloid leukemia is one of the most common malignant hematological and systemic diseases in adults, which is mainly characterized by susceptibility to relapse, poor prognosis, and low survival rate (1, 3, 4). Pyroptosis is a programmed death (5), and RBPs are essential modulators of transcription. Kebin Huang et al. found that MicroRNA-519 enhances HL60 human acute myeloid leukemia cell line proliferation and induces cell apoptosis by reducing the expression level of RBPhuman antigen (18). Additionally, B Mitton et al. reported that a small molecule inhibitor of CREB (cAMP Response-Element Binding Protein), XX-650-23, interaction mostly affects apoptotic, cell-cycle, and survival pathways, which may represent a novel approach for AML therapy (19). However, the relationship between pyroptosis-related RBPgenes and AML remains unclear, so it is important to predict the relationship between pyroptosis-related RBPgenes and AML.

In this study, 71 PRBP DEGs were enriched to cellular protein complex disassembly, RNA catabolic process, RNA transport, RNA destabilization, negative regulation of protein acetylation, ribonucleoprotein complex assembly, and protein export from protein-related pathways such as nucleus. In a study on the expression pattern and clinical value of key m6A RNA modification regulators in abdominal aortic aneurysm, it found that the modified genes were primarily enriched in RNA catabolic process, RNA transport et al. (20). Nelsonet al. demonstrated that a block to efficient splicing can occur at multiple steps in the pathway of normal splicing complex assembly, and plice site selection and ribonucleoprotein complex assembly during *in vitro* pre-mRNA splicing (21). However, the role of the above enriched pathways in AML has not been reported. Stefan Gattenloehner et al. found that the CD56 expression on AML cells correlates with an abnormal expression pattern of runt-related transcription factor 1 (RUNX1) isoforms and the potential for new therapy of CD56(high) AML by suppression of the “overactive” RUNX1/CD56/NF-kappa B signaling pathway(s) (22). Therefore, we guess that certain pathways play an important role in the pathogenesis of AML, and in the future work, we need to further focus our attention on the significance of the enriched pathways such as cellualr protein complex disassembly in AML.

We constructed a risk model of the RBP genes associated with cell pyroptosis in patients with Acute Myeloid Leukaemia. Yi Zhang et al. constructed a novel prognostic scoring model for newly diagnosed FLT3-ITD-positive AML (23), but the model has some limitations such as induction and consolidation treatment regimens cannot be fully harmonized due to the retrospective nature of the study. Yun Wang et al. built an immune risk score to predict survival of patients with AML receiving chemotherapy (24), however, they lacked data on some important predictive covariates, such as mutation topography and results of MRD testing in subjects achieving a complete remission. Piyanuch Kongtim et al. constructed anovel disease risk model for patients with AML receiving allogeneic hematopoietic cell transplantation (25), while this is a retrospective study conducted in a single institution, and the limited number of patients in some subgroups may not detect relevant differences between the groups. Compared with the above models, the risk model we constructed started from the direction of the RBP genes associated with cell pyroptosis. Meanwhile, it had the advantage of fewer model genes.

In constructing this prognostic-related risk model, we obtained a total of six biomarkers. IFIT5, MRPL14, MRPL21, MRPL39, and PUSL1, and none of these genes have yet been reported in AML. MVP encodes the major component of the vault complex. The encoded protein may play a role in multiple cellular processes by regulating the MAP kinase, JAK/STAT and phosphoinositide 3-kinase/Akt signaling pathways. The encoded protein also plays a role in multidrug resistance, and expression of this gene is a prognostic marker for several types of cancer. However, H J Broxterman et al. found that it is shown that Pgp function, but not Mvp/LRP or MRP1 expression correlate with a low steady-state DNR accumulation in *de novo* AML. The Pgp activity does, however, not predict the DNR sensitivity in AML measured as *in vitro* DNR LC50 with an MTT-based assay. The reason for that seems to be that a low DNR accumulation may not be the most important factor in determining the LC50 (26). Therefore, the drug resistance effect of MVP in AML still needs further investigation.

In this study, we performed pathway enrichment analysis between high and low risk groups using GSEA software, and finally analyzed differentially expressed genes between high and low risk groups using the R package limma, and performed functional enrichment analysis of differential genes using the enrichment tool DAVID. Our findings found that the high- and low-risk groups were associated with several immune-related and metabolic-related biological processes and pathways. The high-risk group was associated with activation of the innate immune response and innate immune response activating signal transduction. Curran et al.’ laboratory has recently characterized the host innate immune system generates a T cell tolerant state in an animal AML model (27). Antigen-specific T cell tolerance is a potent immune evasion mechanism in hosts with AML that can be reversed *in vivo* after CD40 engagement (28). These results indicate that immune tolerance to AML may be initiated at the level of the innate immune system (27, 28). Long Zhang et al. discovers antigen-specific T cell tolerance is a potent immune evasion mechanism that can be reversed *in vivo* after CD40 engagement *via* a murine AML model (28). Our findings are consistent with those of the above investigators. Marko Skrtic et al. found that inhibition of mitochondrial translation as a therapeutic strategy for human AML (29). Marvin M van Luijn et al. found that the myeloid leukemic blasts with expressing HLA class II molecules, abnormalities in the processing pathways of endogenous antigens could also result in impaired HLA class II-restricted tumor-associated antigen presentation to CD4(+) T helper cells (30). Hideaki Mizuno et al. revealed that suppression of Fbp1, as well as pentose phosphate pathway enzymes by shRNA-mediated knockdown selectively decreased Evi1-driven leukemogenesis *in vitro*, Considering Evi1 upregulates Fbp1, and supports progression of AML through pentose phosphate pathway activation. Our findings also found that the high-risk group was associated with mitochondrial transport, antigen processing and presentatio and pentose phosphate pathway. Overall, the functional enrichment results for high- and low-risk groups suggested the linkages between RBPs associated with cell pyroptosis and metabolism in AML.

Some limitations of this study also exist, (a) Some functional experiments were needed to further illustrate the underlying molecular mechanisms to predict the role of the cellular pyroptosis-related differential RBP genes in AML; (b) The prognostic model should be validated by more datasets and clinical samples; (c) This study only used bioinformatics methods to conduct multiple analyses based on retrospective data from the public databases, confirmatory experiments *in vivo* and *in vitro* will be required subsequently. Therefore, we will continuously focus on the role of these genes.

In conclusion, through the above analysis, the differential RBP genes related to pyroptosis in AML were screened, and through the regression analysis of these genes, six biomarkers were obtained, and a risk model associated with metabolism was constructed, which provided a theoretical basis and reference value for the future treatment research and prognosis of AML.

## Data availability statement

The datasets presented in this study can be found in online repositories. The names of the repository/repositories and accession number(s) can be found in the article/**Supplementary Material**.

## Author contributions

TB and CL are the principal investigator and conducted statistical analysis and drafted the article. JT and BL performed data management and bioinformatics analysis. TB, CL, F-JL, YW, X-JX, D-JL, JT, and BL edited and revised the article. All authors have read and agreed to the published version of the manuscript.

## Funding

This research was supported by Sanming Project of Medicine in Shenzhen (No.SZSM201911004).

## Conflict of interest

The authors declare that the research was conducted in the absence of any commercial or financial relationships that could be construed as a potential conflict of interest.

## Publisher’s note

All claims expressed in this article are solely those of the authors and do not necessarily represent those of their affiliated organizations, or those of the publisher, the editors and the reviewers. Any product that may be evaluated in this article, or claim that may be made by its manufacturer, is not guaranteed or endorsed by the publisher.

## Supplementary material

Supplementary Figure 1 The PCA and tSNE analyses of patients from high and low risk groups. **(A)** PCA analysis. **(B)** tSNE analysis.

Supplementary Table 1 Gene ontology (GO) analysis on Biological Process (BP).

Supplementary Table 2 Gene ontology (GO) analysis on Cell Components (CC).

Supplementary Table 3 Gene ontology (GO) analysis on Molecular Functions (BP).

Supplementary Table 4 Kyoto Encyclopedia of Genes and Genomes (KEGG) analysis.
