# Supplementary figures and images for: Establishment of a risk model correlated with metabolism based on RNA-binding proteins associated with cell pyroptosis in acute myeloid leukemia
Source: Front Oncol. 2022 Nov 17;12:1059978. doi: 10.3389/fonc.2022.1059978 (PMC9713014; doi:10.3389/fonc.2022.1059978)

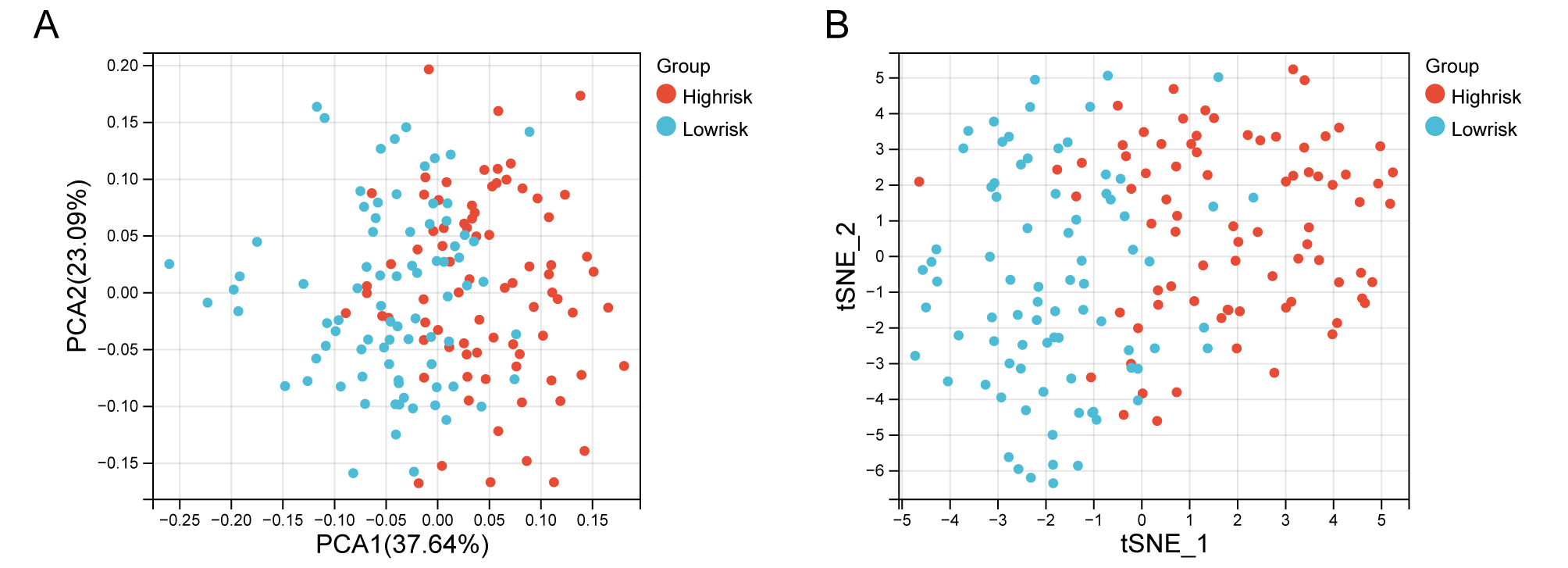

Supplement: Supplementary Figure 1 — The PCA and tSNE analyses of patients from high and low risk groups. (A) PCA analysis. (B) tSNE analysis. [file Image_1.tif]
